# Vibration-Based Measurement of Steel Wire Rope Fundamental Frequency Using an Optical Sensor: Mitigation of Mass-Loading Artifacts

**DOI:** 10.3390/s26216759

**Published:** 2026-10-26

**Authors:** Lukáš Timko, Pavel Peterka

**Affiliations:** Institute of Logistics and Transport, Faculty of Mining, Ecology, Process Control and Geotechnology, Technical University of Košice, Park Komenského 14, 042 00 Kosice, Slovakia; lukas.timko.3@student.tuke.sk

**Keywords:** steel wire rope, optical flow sensor, vibration analysis, fundamental natural frequency, metrological sensitivity, mass-loading effect, tension estimation

## Abstract

**Highlights:**

**What are the main findings?**

- Optical method avoids mass-loading and local stiffening errors of contact sensors.
- A 28 g target plate mitigates helical surface artifacts without altering stiffness.

**What are the implications of the main findings?**

- Repeatability across ten independent ropes yields a combined uncertainty of 0.0106 Hz.
- Controlled wire cuts establish a laboratory sensitivity limit (1.35% area loss).
- Isolates dynamic responses for baseline verification under controlled conditions.

**Abstract:**

Non-destructive testing and tension estimation of static steel wire ropes using vibration analysis are conventionally distorted by deterministic mass-loading and local bending stiffness alterations introduced by rigidly clamped contact accelerometers, which artificially shift the system’s dynamic baseline. To address this measurement error, a minimally invasive optical methodology utilizing a 25,600 DPI optical flow sensor architecture is proposed. The method’s metrological validity was verified against a theoretical beam-string model—incorporating the effective rope modulus ($E_{\mathrm{eff}}$) and structural geometric factor ($G_{m}$)—and contact accelerometer measurements across ten independent 6×37 FC steel rope specimens tensioned up to 64 kN. By eliminating the 191 g mass-loading and clamping-stiffness artifacts of traditional accelerometers (validated on a 0 g bare rope surface) and utilizing a lightweight 28 g reflective target plate during degradation tests to mitigate helical surface artifacts without altering local bending stiffness, the measurement chain achieved a combined standard uncertainty of $u_{c}=0.0106\;\mathrm{Hz}$ (expanded uncertainty U=0.0212 Hz). To establish the laboratory sensitivity limit of the optical chain, progressive structural degradation was artificially induced across all ten specimens by severing 3 to 35 wires at mid-span under constant tension. The system reliably detected initial damage at 3 broken wires (1.35% loss of metallic cross-section, causing a 0.1 Hz shift at 16 kN) and resolved incremental frequency shifts down to Δf=0.036 Hz (between 3 and 5 broken wires, exceeding the $3u_{c}=0.0318\;\mathrm{Hz}$ significance threshold). While these artificial wire cuts represent a laboratory metrological sensitivity limit rather than a universal field NDT calibration, the linear correlation at 64 kN ($R^{2}=0.9419$) confirms that localized axial stiffness loss dominates the dynamic response. The proposed optical approach provides a reliable framework for laboratory baseline verification and tension assessment under controlled conditions.

## 1. Introduction

In terms of construction, steel wire rope is a complex structure of wires that exhibits high axial strength and good bending flexibility. These mechanical properties make wire ropes suitable for a wide range of industrial and transport applications. On the other hand, the complexity of wire rope construction poses a challenge to the process of monitoring its durability and integrity in service. Monitoring the condition of wire ropes on industrial and transportation equipment is crucial for the safety of their operation and health protection of transported persons or operators [1,2,3,4]. According to Zhang et al. [5,6], the load on the wire ropes in service, lack of maintenance, climatic and working conditions (high temperature, aggressive environment) have an influence on the formation of specific wire rope defects. Monitoring the condition of wire ropes in service is addressed by means of non-destructive testing (NDT) methods [7,8]. The most widely used methods for monitoring the integrity of wire ropes include magnetic flux leakage (MFL) [9]. This electromagnetic NDT method allows the detection of local internal and external wire rope breaks (LF) [10,11] and monitoring of changes in the wire rope cross-sectional metallic area (LMA) [12,13] caused by corrosion or abrasion of the rope in service. The MFL methodology that has been known for more than 100 years cannot cover all the specifics of defects occurring in wire ropes. The MFL method is only applicable to moving ropes or to ropes where it is possible to move the NDT device smoothly. Another limitation of the MFL method is high sensitivity to rope vibration according to Liu and Ren [14] and electromagnetic interference and magnetizing properties of the steel grade from which the wire rope is made according to Yan et al. [15]. A number of authors report methodologies to correct the above-mentioned shortcomings of the MFL method in NDT of moving wire ropes [16,17,18,19,20].

Static ropes, ropes with poor access, bridge ropes, anchor ropes, and rope to terminal transition points are challenging when searching for new NDT techniques and methods. In the literature, one can encounter the application of practically all known NDT methods. Gamma rays were applied by Peng and Wang [21] to the inspection of steel cables of suspension bridges due to the inaccessibility of the cables for visual or other NDT methods. The use of eddy currents for non-destructive and quantitative evaluation of steel cables proposed by Cao et al. [22] is based on the radial function of the neural network. Thermal measurements were used by Battini et al. [23] to predict the fatigue life of steel wire ropes. Among the methods beyond the standard NDT methods, the use of commercial optical fibre to detect wire breaks on the wire rope was proposed by Cortázar et al. [24].

A separate group of NDT methods is made up of principles that exploit mechanical properties of the steel (or textile rope) such as its elasticity (i.e., flexibility and bendability) [25,26,27,28,29]. The string-like behaviour of the wire rope has inspired a number of authors to use guided ultrasonic waves, acoustic emission and various applications of vibration detection to monitor the rope condition. The use of ultrasonic guided waves (USGW) was addressed in papers [25,30,31,32,33], alongside approaches determining wire rope condition via acoustic emission [34,35,36].

The relationship between the characteristics of the acoustic emission signal and the wire breakage rate of the wire rope is given by the energy and amplitude according to Drummond et al. [34]. The advantage of using acoustic emission is the possibility of applying the method to wire ropes made of artificial or natural textile fibres according to Li et al. [37,38]. From the combination of methods, Vanniamparambil et al. [39] published a method based on digital image correlation of optical images, guided ultrasonic waves, and acoustic emission for the detection of broken wires.

Huang et al. [40] studied the surface damage detection for steel wire ropes using deep learning and computer vision techniques. Digital image correlation (DIC) and vision-based methods [38,40] provide full-field information regarding structural displacement and strain mapping.

Xu et al. [41] proposed a novel approach for cable tension estimation and monitoring via the mode shape method. They established a refined cable model considering various boundary conditions; subsequently, the dynamic stiffness method was used to calculate the variations in the sine and hyperbolic sine (including hyperbolic cosine) components of the cable mode shape under various operational states.

Vibration use to determine the condition of wire ropes can be first observed in the work of Kwun and Burkhardt [42,43]. They followed the work of Winter and Green [44] (1985), who used mechanical spectroscopy to monitor the condition of textile ropes (fibre ropes). The authors monitored characteristics of the wave development in a tensioned rope. The methodology was able to distinguish a new rope from a used one. Ferreira et al. [45] studied the textile rope dynamic modulus using longitudinal wave propagation and described the rope elastic properties. By measuring its velocity and at a known rope density, the authors determined the dynamic modulus that served as an indicator of the rope condition. The research results of Kwun and Burkhardt [42,43], Winter and Green [44] and Ferreira et al. [45] were extended to the NDT method of laser non-contact scanning vibrometry. Laura et al. [46] described the use of infrared emitting and receiving diodes to monitor the vibration of steel and textile rope, respectively. Padilla et al. [47] (2015) patented a non-contact NDT solution for textile rope. Laser Doppler vibrometry (LDV) and related non-contact optical scanning methods [42,43,44,45,46,47] avoid contact mass loading and are powerful for remote vibration measurement, yet they typically require higher cost, careful optical alignment, and favorable surface reflectance/access.

Zhu et al. [48] used a thin rod FBG vibration sensor for steel wire rope monitoring. The measurement was focused on recording of the steel wire rope cracking pulse. Different variations in the FBG vibration sensor were used on monitoring of steel wire ropes vibrations by Yu et al. [49], Zhang et al. [50] and Velazquez-Carreón et al. [51]. Shao et al. [52], Wang et al. [53], Li et al. [54], Zhu et al. [55], and Liu et al. [56] used a Fiber Bragg acceleration sensor for monitoring of low vibration.

Despite the availability of various NDT and vibration monitoring techniques, a critical research gap remains regarding the highly sensitive, non-intrusive monitoring of tensioned steel wire ropes without physically altering their dynamic baseline. Contact methods, such as MFL or FBG sensors [48,49,50,51,52,53,54,55,56], are inherently intrusive or require physical coupling that can introduce mass-loading and stiffness alterations. Conversely, while non-contact full-field methods like DIC and vision-based techniques [38,40] provide extensive data, they are inherently limited by camera resolution, lighting conditions, and prohibitive computational costs when resolving minute frequency shifts on the order of hundredths of a Hertz. Similarly, although LDV [42,43,44,45,46,47] offers extreme precision without mass-loading, it is typically highly expensive, requires careful optical alignment, and is heavily dependent on favorable surface reflectance. To bridge this gap, the proposed methodology utilizes a high-resolution optical flow sensor (HERO 25K). This approach provides a strictly non-contact, cost-effective, and computationally lightweight alternative. It must be noted that while this method is highly effective for evaluating rope tension and static conditions in controlled laboratory or industrial environments, assessing its on-site feasibility for field scenarios—such as bridge or anchor cables subjected to wind-induced vibrations and severe environmental drift—remains outside the scope of this baseline metrological validation.

While contact accelerometers are the established industry standard for vibration monitoring, their direct application to relatively lightweight, highly tensioned steel ropes introduces specific physical challenges. The necessity of rigid attachment to the rope surface inevitably introduces a local discontinuity. Specifically, the inherent mass of the contact sensor assembly generates a deterministic mass-loading effect, while the clamping mechanism locally alters the bending stiffness of the wire rope. In macroscopic structural monitoring, this physical interference may be negligible. However, when the objective is to track minute shifts in fundamental natural frequencies (in the order of hundredths of a Hertz) to identify early-stage structural degradation, this deterministic alteration of the dynamic baseline becomes a limiting factor. To address this measurement challenge without compromising the natural dynamics of the tensioned rope, this article presents the application of a minimally invasive optical methodology. By utilizing a high-resolution optical flow sensor, the proposed approach isolates the pure dynamic response of the steel wire rope. This eliminates the mass-loading artifact associated with contact methods, providing a reliable alternative for high-precision diagnostic measurements where altering the system’s baseline mass and stiffness is undesirable.

The objective of the presented measurements and analyses is to introduce a minimally invasive optical method and demonstrate its metrological sensitivity through the following procedures:

- verification of the method’s sensitivity to changes in rope tension/stiffness,
- verification of the method’s sensitivity to local changes in rope mass (simulating the effect of attached contact vibration sensors on the natural frequency),
- verification of the method’s sensitivity bound using artificially induced local wire cuts.

## 2. Materials and Methods

**Figure 1:**
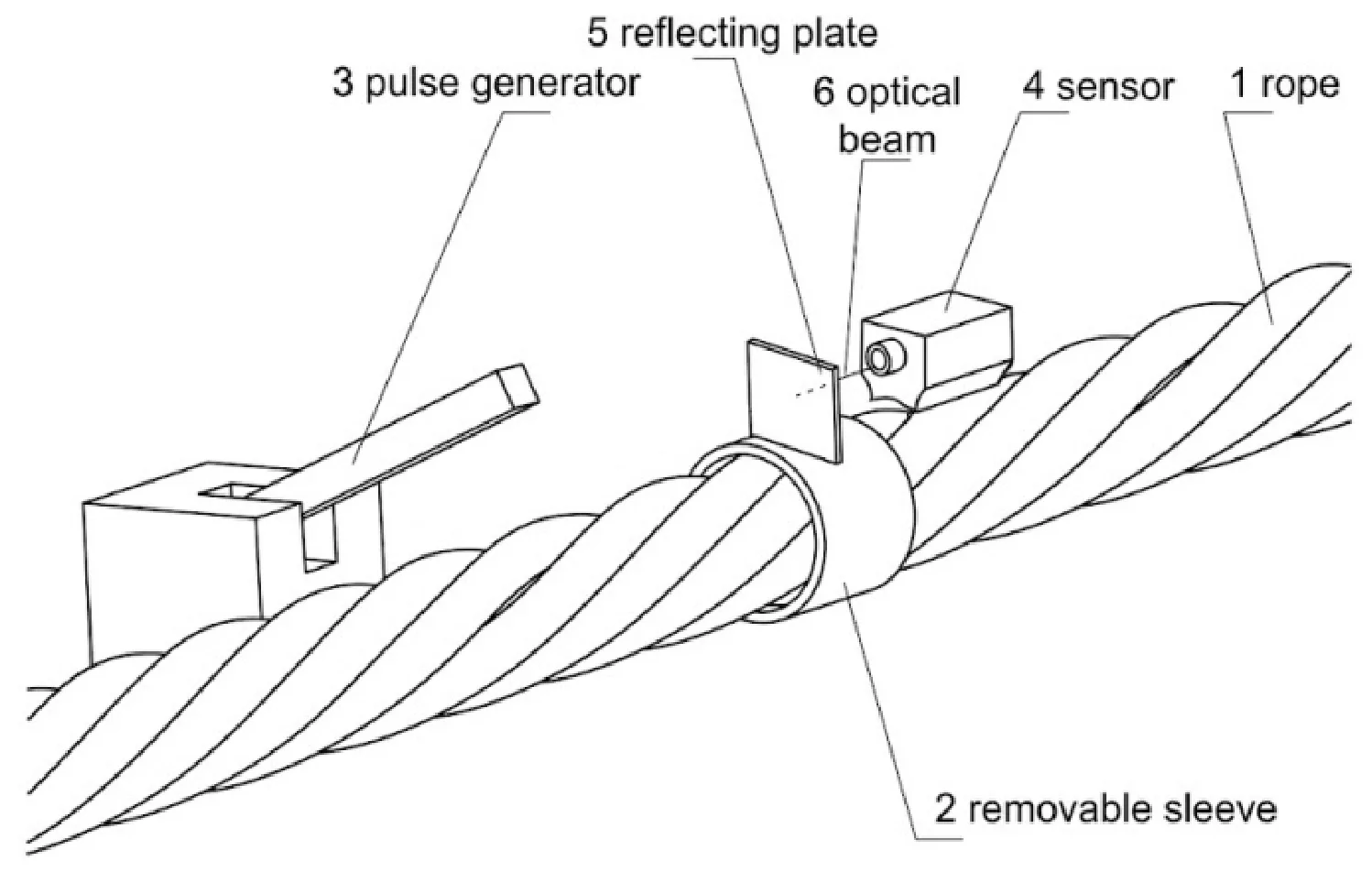
Schematic diagram of the optical device.

The optical device consists of a rope (Figure 1 position 1) on which a removable sleeve (Figure 1 position 2) containing one reflecting plate (Figure 1 position 5) is mounted. A pulse oscillation generator (Figure 1 position 3), which is located outside the tensioned rope, ensures that it is always oscillated by the same excitation force. A sensor (Figure 1 position 4) is positioned outside the tensioned rope (Figure 1 position 1) so that its optical beams (Figure 1 position 6) hit the reflecting plate (Figure 1 position 5) and record the rope oscillation forced by the pulse oscillation generator (Figure 1 position 3). For baseline monitoring of the rope’s dynamic response, the frequency characteristics (Figure 1, position 1) established during initial measurements serve as the reference state.

**Figure 2:**
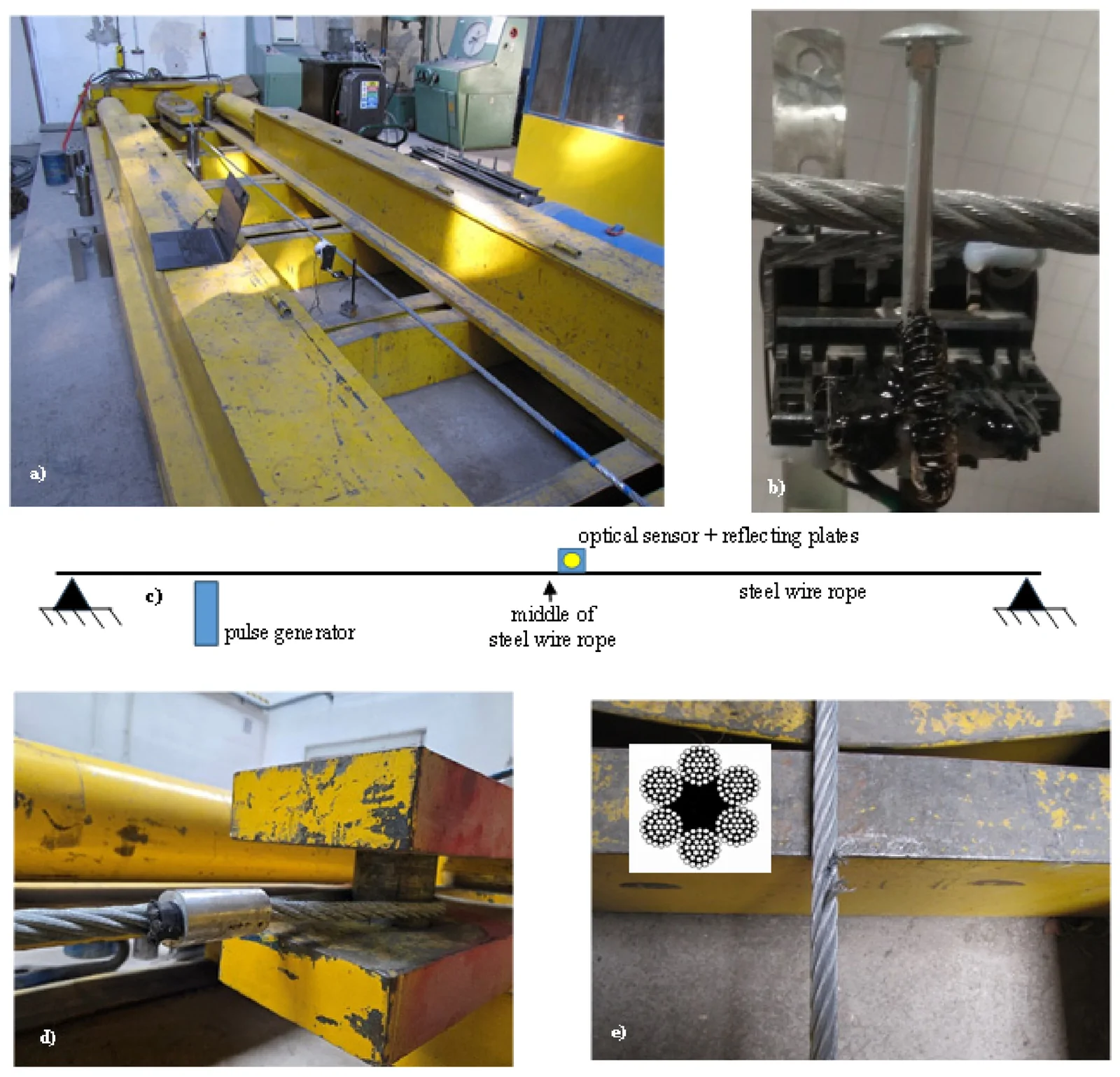
Test equipment and steel wire rope sample. (**a**) View of the optical sensor placement; (**b**) Pulse generator; (**c**) Layout of the measurement setup components; (**d**) Method of securing the steel rope in the tensile testing machine; (**e**) Artificially induced local damage to the steel rope, cross-section of the test specimen, rope construction 6×37M FC.

Based on practical measurements of anchor ropes (guy wires) for water towers, transmitter towers, and chimneys, a rope construction of 6×37M FC 1570 U sZ (Table 1, Figure 2e), commonly used in our region (Slovakia), was selected for the experiments. The steel rope test specimens are terminated with a ferrule-secured eye for anchoring within the measurement system (Figure 2d). For testing purposes, a total set of ten rope specimens was prepared from a single rope drum to guarantee identical structural and material properties. The effective vibrating length of each specimen, defined as the free span between the securing ferrules, was 5.5 m. These specimens were tensioned in a tensile testing machine (Figure 2a,c) under a controlled tensile force. All ten specimens were independently subjected to the full experimental protocol—including the progressive wire-cutting degradation tests—to ensure rigorous statistical repeatability across multiple physical ropes.

**Table 1:** Rope Parameters Used for Testing.

Nominal Rope Diameter [mm]	Nominal Wire Diameter [mm]	Nominal Metallic Cross-Sectional Area [mm^2^]	Tensile Strength Grade [MPa]	Calculated Minimum Aggregate Breaking Force [kN]	Minimal Breaking Force [kN]	The Rope Length Density [kg·m^−1^]	Effective Rope Modulus ($E_{\mathrm{eff}}$) [Pa]
18.0	0.8	111.6	1570	175.2	147.51	1.03	*

* The analysis of the Effective rope modulus ($E_{\mathrm{eff}}$) is presented in Section 2, Table 2.

**Table 2:** Values of Effective rope modulus ($E_{\mathrm{eff}}$) and experimental data *F*(Δ*L*).

Force *F* [kN]	Average Rope Elongation Δ*L* [mm]	Calculated Effective Rope Modulus ($E_{\mathrm{eff}}$) [Pa]
4	13.87	starting point (rope settling)
8	8.28	≈0.85.10^11^
16	10.67	≈1.12.10^11^
32	16.76	≈1.35.10^11^
64	20.21	≈1.62.10^11^

The sets of measurements were conducted to verify the method’s sensitivity to changes in the stiffness of the string-like steel rope. A real steel rope (Table 1) was progressively tensioned at forces of 4 kN, 8 kN, 16 kN, 32 kN, and 64 kN. These selected values represent 2.7%, 5.4%, 10.8%, 21.7%, and 43.4% of the rope’s minimum breaking force (Table 1), respectively. The anchoring of the steel rope to the tensile testing machine (Figure 2d) corresponds to Case 1—support on both rope sides (Section 3). In physical reality, however, the ferrule-secured eye and pin termination introduces a localized bending layer that slightly shortens the uncalibrated effective vibrating length compared to an ideal pin support, contributing to the measured frequencies systematically exceeding the theoretical Case 1 values.

To verify the measured fundamental natural frequencies of the actual steel rope, theoretical values were calculated using the beam-string model (Equation (5), Section 3), which incorporates the geometric factor *G_m_* determined for the specific 6×37 FC construction. Calculating this theoretical curve required determining the Effective rope modulus ($E_{\mathrm{eff}}$) of the tested rope at each tension level. Rope elongation (Δ*L*) was recorded after the specimen had dynamically settled through ten tensioning cycles (from 0 to 50% *F_min_*). The initial elongation of 13.87 mm at a 4 kN tension was attributed primarily to the constructional settling of the fibre core. Therefore, the measurement system baseline was established at this 4 kN point. The elongation values recorded at subsequent higher forces (Table 2) represent the structural stretch of the consolidated rope, from which the specific Effective rope modulus ($E_{\mathrm{eff}}$) for each load step was calculated and subsequently applied in Equation (5).

Measurements establishing the sensitivity bound aimed to evaluate the optical method’s ability to detect minute frequency shifts caused by local reductions in the metallic cross-section. The change in cross-section was induced by the progressive creation of artificial wire breaks (Figure 2e). To maintain strict methodological consistency, the rope was never unloaded during the degradation process. The disruptions were performed directly under load, with the testing machine continuously maintaining the constant 64 kN tension to prevent any global structural relaxation, macroscopic unwinding, or hysteresis effects. Specifically, the damage was induced exactly at the mid-span of the effective length, aligning with the antinode of the fundamental oscillation mode to maximize the system’s sensitivity to stiffness changes. All cuts were concentrated at this single cross-section within one specific outer strand to simulate a severe macroscopic local fault (LF).

In the center of the rope, 3, 5, 10, 15, 20, and 25 breaks were created. The local failure of the strand wires resulted in a metallic cross-section reduction of 3 breaks—1.35%, 5 breaks—2.25%, 10 breaks—4.5%, 15 breaks—6.76%, 20 breaks—9.01%, and 25 breaks—11.26%. At a tension force of 64 kN, the measurements were extended to include 30 and 35 wire breaks, representing a reduction in the metallic cross-section of 13.5% and 15.8%, respectively.

All experiments were conducted in a temperature-controlled hall at a constant temperature of 18 °C.

The measurement procedure consisted of the following steps:

- installation of the steel rope specimen into the tensile testing machine;
- tensioning the rope to the selected force (continuously monitored throughout the process);
- for verification measurements of the mass effect on the resulting natural frequency: attaching and exchanging weights on the tensioned rope;
- for sensitivity bound verification: creating wire cuts on the tensioned rope;
- positioning the optical sensor;
- software startup and calibration;
- inducing vibrations using the pulse generator;
- recording of the measurement data for 30 s;
- data evaluation and storage.

Specific components of the measurement setup include the pulse generator (Figure 1, pos. 3) and the reflective plate (Figure 1, pos. 5). The impact is generated by an electric coil pulse generator, ensuring consistent impact energy throughout the measurement series. No damage to the rope specimen by the pulse generator hammer was observed.

The use of a reflective plate is not a limiting factor for vibration measurements. To ensure strict methodological correctness, the optical measurements were divided into two stages utilizing different sensing targets. First, for the fundamental verification against the theoretical models (Equations (1) and (5)) and the 191 g accelerometer assembly, the measurements were performed without the plate (0 g added mass), sensing directly from the bare rope surface coated with black spray paint. This guaranteed completely uninfluenced natural frequency. Second, for the high-precision sensitivity bound measurements, a lightweight flat reflective plate (mass 28 g) was rigidly attached to the rope. The implementation of this reflective plate was also essential to overcome the inherent hardware limitations of the HERO 25K optical flow sensor. Specifically, it eliminated optical artifacts generated by the helical surface geometry of the rope and ensured that the tracked surface remained within the sensor’s narrow focal depth (lift-off distance) during complex 3D oscillations. The verification of the optical sensor was performed by calculation according to the theoretical model (Section 3) and by comparing the values obtained through simultaneous measurement using accelerometers. Two KAS903-12A accelerometers, attached to the steel rope using a tight anchoring fixture, were used for the comparative measurements.

### 2.1. Technical Specification of the Measurement System

The measurement apparatus integrates an optical correlator based on the HERO 25K architecture (Table 3). The optical sensor features a native resolution of 25,600 DPI without digital upscaling, which directly minimizes measurement uncertainty.

Importantly, chip DPI and the nominal 0.992 µm step must not be equated with end-to-end displacement accuracy. Actual accuracy depends on lens depth of field, surface reflectance/speckle, defocus, motion blur, and out-of-plane (3D) oscillations. Prior to dynamic experiments, the sensor underwent static spatial calibration on a micrometer linear stage to verify linearity under controlled standoff; the linearity error was less than 1.0% when the absolute lift-off was maintained. During all subsequent measurements, lift-off was physically constrained to 1.5 mm ± 0.2 mm by a rigid sensor mount. For plate-based tests, the flat reflective target further helped keep the tracked surface within this narrow focal window during small-amplitude transverse oscillation. A full formal metrological traceability chain and dedicated long-term thermal/time-drift characterization were outside the present scope and are listed as limitations for future work. Sampling integrity was monitored in software (Fs-threshold warning below 450 Hz). Furthermore, the temporal stability of the 1000 Hz USB HID polling rate and sampling timestamps was continuously verified in the acquisition script using high-resolution system performance counters to ensure deterministic 1.0 ms intervals without packet drops. To eliminate deterministic signal distortions inherent to commercial HID controllers, internal hardware acceleration, data-smoothing algorithms, and trajectory prediction functions (e.g., angle snapping) were disabled at the firmware level, so that recorded raw optical-flow data represent the physical displacement of the oscillating target.

### 2.2. Hardware and Software Specifications for Measurement Recording and Evaluation

The parameters required for data acquisition were determined based on the theoretical calculation of the expected natural frequencies for the specific steel rope (Table 1) at the predefined tension levels mentioned above. The range of measured fundamental natural frequencies was anticipated to be between 5 Hz and 25 Hz. Generally, it is necessary to monitor 3 to 5 times the fundamental frequency [49].

The displacement data was acquired at a sampling frequency of f_s_ = 1000 Hz. The data acquisition was managed via a custom-developed Python script (Python 3.13.3, with NumPy 2.2.6 and SciPy 1.15.3) over 30-s measurement windows. To isolate the fundamental natural frequencies and remove mechanical drift, the raw signal was filtered using a 5th-order Butterworth bandpass filter (1 Hz–200 Hz). Zero-phase filtering was applied to prevent phase distortion in the acquired data.

**Figure 3:**
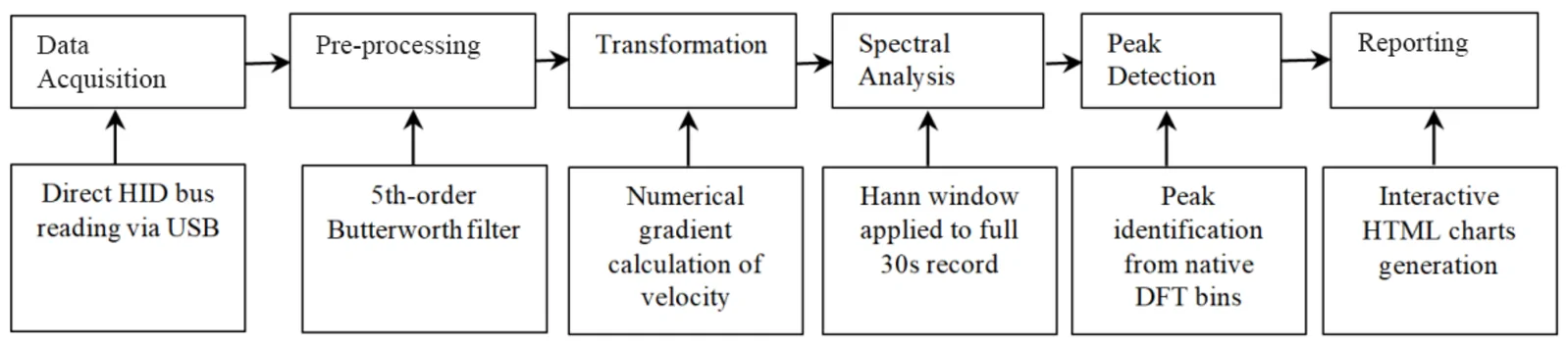
Block diagram of signal processing.

A standard Fast Fourier Transform (FFT) of the full 30-s record (30,000 samples) yields a native frequency bin resolution of Δf = 1/T ≈ 0.033 Hz. To preserve this high physical resolution, the spectral estimation was performed using a single Discrete Fourier Transform (DFT) across the entire unsegmented record, applying a Hann window to mitigate spectral leakage. Because the native bin width (0.033 Hz) is smaller than the smallest identified frequency shift (0.036 Hz), the system physically supports the detection of these minute baseline deviations directly, without relying on windowed averaging methods or sub-bin interpolation artifacts. Peak identification was performed by detecting the discrete spectral maximum of the fundamental frequency bin. No sub-bin interpolation (such as parabolic or sinusoidal fitting) was applied, ensuring the observed frequency shifts directly reflect the native spectral discretization. This direct bin-maximum identification was reliable due to the high signal-to-noise ratio (SNR) achieved via controlled electromagnetic pulse excitation and flat-plate optical tracking, where the fundamental spectral peaks exceeded the baseline noise floor by more than an order of magnitude, and no USB frame loss (monitored via the 450 Hz threshold) occurred during the 30-s acquisition windows (Figure 3, Table 4).

## 3. Theoretical Information

The following assumptions were made to model the ideal rope oscillation process [57,58,59,60,61,62,63]:

- Wire rope is perfectly flexible and during bending the elastic deformation energy of the rope will be negligible with respect to the total energy of the rope oscillation;
- Wire rope is tight between two points through its ends by a force *F* and can oscillate motion;
- In the case of small rope oscillations, each rope length segment *dl* performs harmonic oscillations about its equilibrium position (the rope position is at rest);
- Due to the oscillations, we neglect the rope elongation, i.e., each point on the rope is stretched by the same force *F*;
- The influence of the gravitational field is negligibly small (during oscillation the change in the rope element potential energy is negligibly small).

### Fundamental Natural Frequency of an Ideal Rope

The natural angular frequency ω is determined from the boundary conditions, i.e., the conditions describing the behavior of the steel rope at its anchor points [63]. For a rigidly clamped rope, we assume zero displacement at the points of attachment. For a tensioned rope of length *L*, the following applies:

(1) $$\omega_{n}=\frac{n\pi }{L}\sqrt{\frac{F}{\mu }}\;,\;\mathrm{or}\;f_{n}=\frac{n}{2L}\sqrt{\frac{F}{\mu }}$$

where: *ω_n_*—natural angular frequency [rad·s^−1^];

*F*—steel rope tension force [N];

*µ*—nominal mass of the steel rope [kg·m^−1^];

*n* = 1, 2, 3…, harmonic frequency number;

*f_n_*—fundamental natural frequency of the rope [Hz].

Natural oscillations with the lowest frequency are also referred to as the fundamental frequency *f*_1_. Subsequent natural frequencies are referred to as higher harmonic frequencies.

The geometric factor *G_m_* analysis assumes high friction between the strands and the individual wires in the rope structure. The assumption was confirmed by experimental measurements where the actual value of the geometric factor *G_m_* is close to minimum slip between wires and strands in the rope structure.

**Figure 4:**
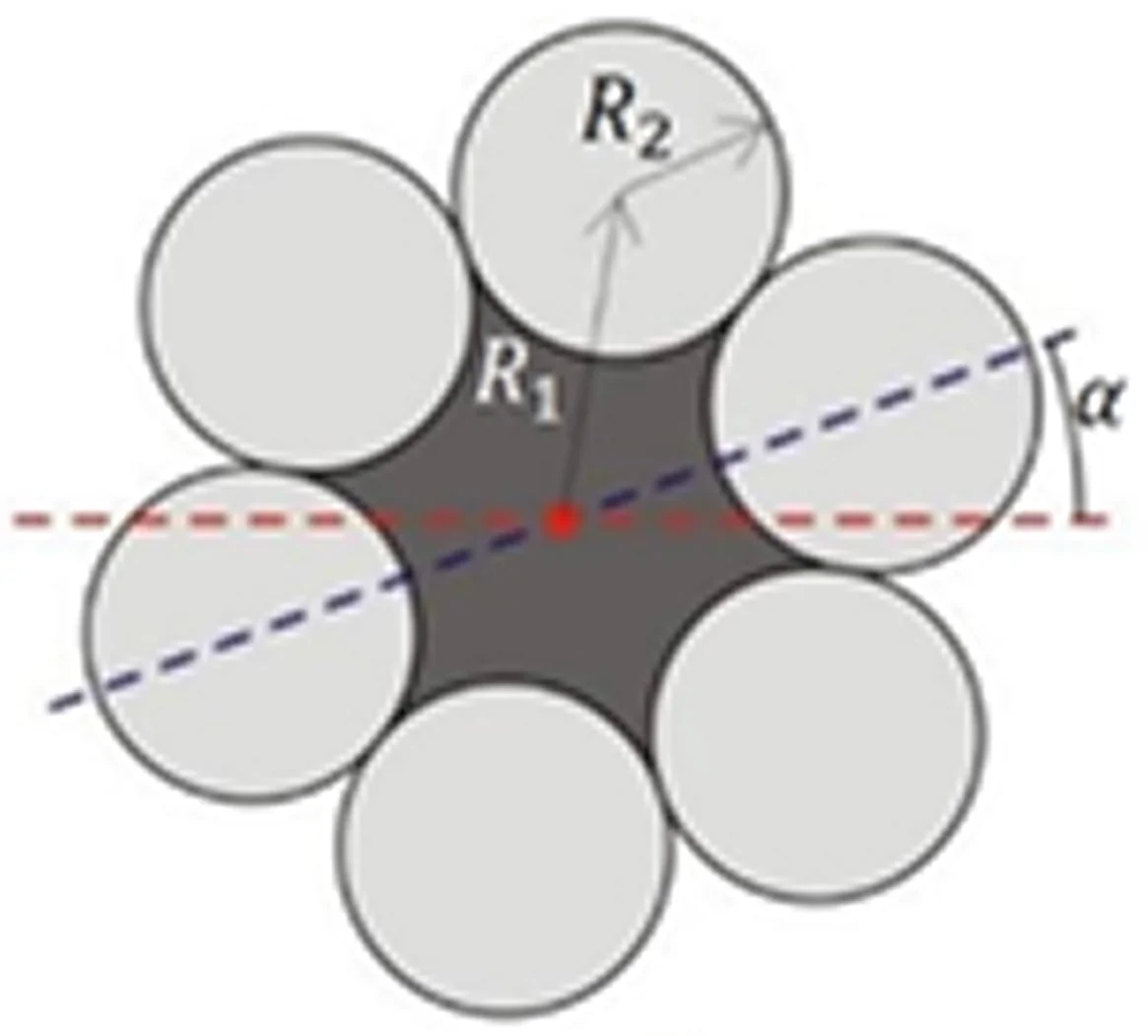
Six-strand steel wire rope with textile core.

The middle part of the 6 × 37 six-strand steel rope (Figure 4) is filled with a textile cord that has no elastic properties. We assume that the textile cord acts on the induced oscillations in the rope only by its inertia. The steel rope consists of six strands with radius *R*_2_, the midpoint of which is at the distance *R*_1_ from the rope midpoint (Figure 4). A strand of the wire rope is wound helically around the rope cord, and its position is twisted around the rope midpoint by an angle α (Figure 4).

By adjusting for the whole rope cross-section (6 strands), we obtain the equation for calculating the geometric factor of a six-stranded rope:

(2) $$G_{m}=\frac{3\pi }{2}R_{2}^{2}\left(2R_{1}^{2}+R_{2}^{2}\right),$$

here: R_1_ = 2R, R_2_ = R (Figure 4), rope diameter D = 2(R_1_ + R_2_), D = 6R.

For the tested six strand steel wire rope 6 × 37 (222 wire rope) (Table 1, Figure 4), we can express the geometric factor *G_m_*:

(3) $$G_{m}\approx 0.024D^{4},$$

where *G_m_* represents the effective cross-sectional moment of inertia [m^4^] for the specific 6 × 37 geometrical arrangement.

In a real wire rope application, we assume three ways of attaching the rope ends, three boundary conditions [60,62]:

- case 1. both rope ends are supported (Track ropes of aerial ropeways),
- case 2. one rope end is supported, and the other one is embedded (Stay ropes, Tensioning ropes),
- case 3. both rope ends are embedded (Stay ropes).

The anchoring of the steel rope to the tensile testing machine (Figure 2d) corresponds to Case 1—support on both rope sides.

The natural circular oscillation frequency of the steel wire rope ω_n_ is defined as (in the SI system):

(4) $$\omega_{n}=\frac{m_{1,n}}{L}\sqrt{{\left(\frac{m_{1,n}}{L}\right)}^{2}\frac{EG_{m}}{\mu }+\frac{F}{\mu }}\;\mathrm{or}\;\omega_{n}=\frac{\pi n}{L}\sqrt{{\left(\frac{\pi n}{L}\right)}^{2}\frac{EG_{m}}{\mu }+\frac{F}{\mu }}$$

then the natural oscillation frequency of the steel wire rope $f_{n}$ is expressed as:

(5) $$f_{n}=\frac{n}{2L}\sqrt{{\left(\frac{\pi n}{L}\right)}^{2}\frac{EG_{m}}{\mu }+\frac{F}{\mu }}$$

From Equation (5) the following applies:

- if the steel wire rope is stretched by a very large force ${\left(\frac{\pi n}{L}\right)}^{2}EG_{m}\ll F$ the fundamental natural frequency of the wire rope oscillation reaches the fundamental natural frequency of the ideal rope oscillation. The change occurs only for higher harmonic frequencies and with increasing value n,
- for very high harmonic frequencies or poorly stretched steel wire rope ${\left(\frac{\pi n}{L}\right)}^{2}EG_{m}\gg F$, the wire rope oscillates similarly like a rod, and higher harmonic frequencies are proportional to *n*^2^.

In general, it is necessary to find a numerical solution for specific values of physical parameters such as *E*, *G_m_*, *ρ*, *F*, *S*, *L*, which express the properties of a particular steel wire rope. Due to its construction, a steel wire rope represents a mechanically complex structure. For this reason, when defining its stiffness, the steel rope must be described as a system with variable effective bending stiffness. Changes in bending stiffness can be induced by variations in the tension force; specifically, as the tension force increases, the steel rope increasingly behaves like a pre-stressed string. An ideal rope model represents a system with zero bending stiffness. In a real rope, inter-wire slippage occurs during oscillation, which is gradually restricted as the tension force increases. This leads to a gradual ‘locking’ of the rope structure. As the wires are pressed together, the relative motion caused by friction is increasingly limited, and the rope’s behavior converges toward a theoretical model that incorporates the geometric factor. The theoretical model of an oscillating steel rope, including the geometric factor *G_m_*, accounts for the rope’s cross-section and assumes maximum friction between its individual wires. The geometric factor should not be viewed as constant, but rather as a target state where wire slippage during vibration is prevented, depending on the applied load.

## 4. Verification Process of the Optical Measurement

The verification of the optical measurement of the rope’s natural frequency of vibration was conducted through a comparative analysis in three steps, utilizing mathematical models (Equations (1) and (5)) and contact measurement via an accelerometer as reference baselines. The validation process of the optical data consists of the physical interpretation of the deviations between these three approaches.

### 4.1. Establishing the Theoretical Reference Baseline

In the first step, the theoretical frequency framework was established using Equation (5) considering an ideal pinned support (Case 1, Section 3). The difference between the models in Equations (1) and (5) lies in the utilization of the 6×37 FC steel wire rope properties, specifically the Effective rope modulus ($E_{\mathrm{eff}}$) and the Geometric factor *G_m_*. Equation (1) serves to verify the initial state (baseline), while the degradation is purely a matter of local stiffness variation, which the classic string equation without corrections does not describe.

**Figure 5:**
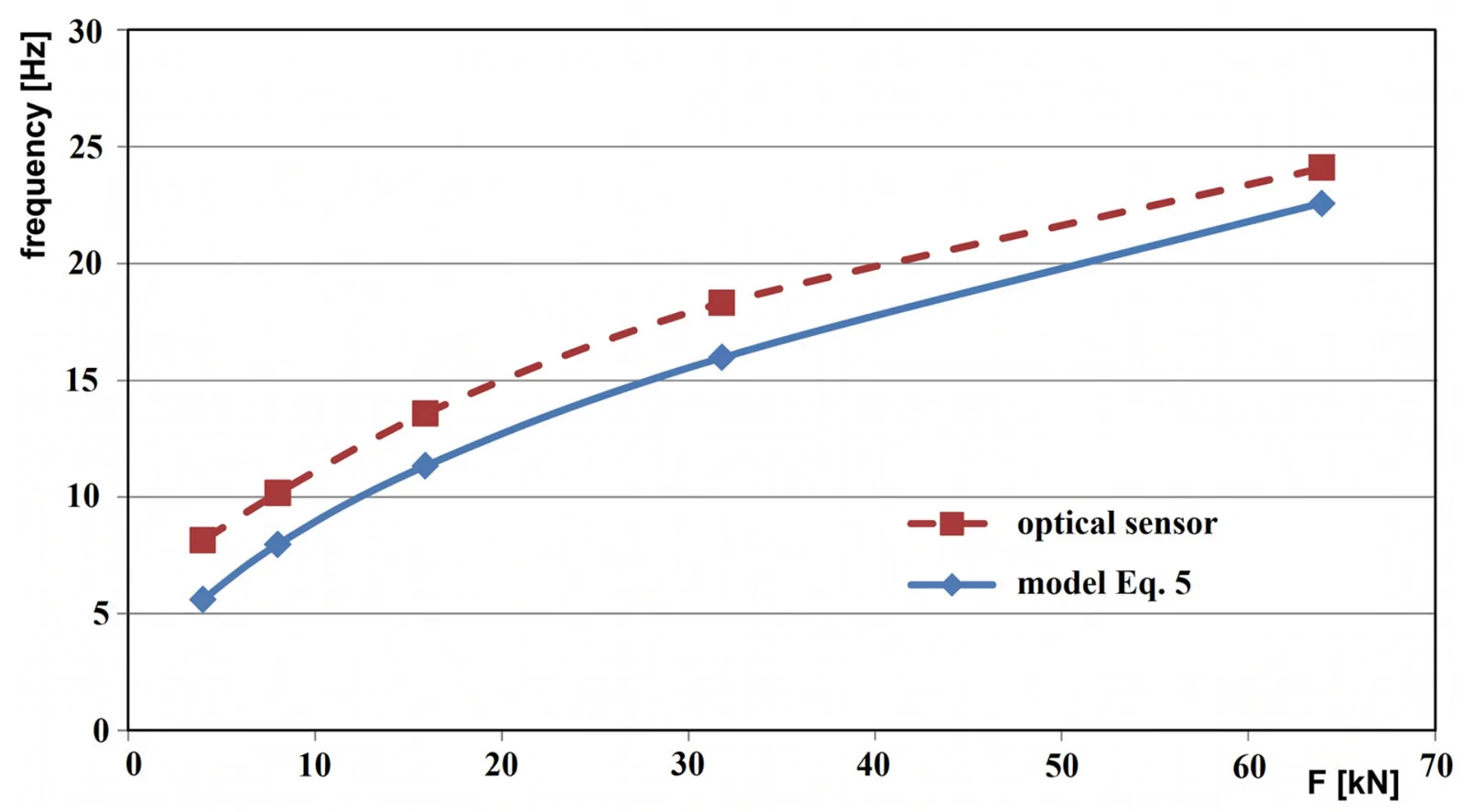
Comparison of natural frequency trends measured by the optical sensor vs. calculated using Equation (5) at rope tension forces F: 4 kN, 8 kN, 16 kN, 32 kN, and 64 kN. Measurements performed using the bare rope surface (0 g added mass).

Figure 5 displays a comparison of the natural frequency trends measured using the optical sensor (red dashed line) and the real rope model calculated using Equation (5) (blue line). The natural frequency values measured by the optical sensor exhibit higher values, which is caused by the influence of real boundary conditions related to the rope anchoring (Case 1). The termination of the rope (eye with a pressed ferrule) and the anchoring of the rope (on a pin) into the tensile testing machine create boundary bending layers during the vibration process, thereby shortening the effective oscillating length of the rope. The result of shortening the effective oscillating length of the rope is a proportional increase in the natural frequency of the steel wire rope. Figure 5 shows two parallel curves with the same mathematical trend, with an upward shift in the real natural frequency values of the rope measured by the optical sensor. The functional course of both curves thus confirms the well-known theory regarding the increase in the natural vibration frequency proportional to the square root of the tension force. Although highly precise tension estimation is a primary application of the proposed optical method, an independent numerical calibration of the exact effective length (*L_eff_*) was not strictly required for the purpose of the structural degradation tests. As observed in Figure 5, the boundary bending layers introduced by the anchoring eyes create a systematic offset between the theoretical model and the actual measurement. However, during the progressive wire cutting at a constant 64 kN tension, these specific boundary conditions remained entirely constant. Therefore, their influence acts as a stable systematic baseline offset that does not invalidate the metrological measurement of minute relative frequency changes (Δ*f*) caused by the induced local defects. Naturally, for absolute tension estimation in future field applications, an empirical calibration of *L_eff_* specific to the actual anchoring conditions will be necessary.

### 4.2. Quantification of the Contact Method Influence Using an Accelerometer

In the second step, a comparative measurement was performed using a contact accelerometer with a total apparatus mass of 191 g (2× accelerometer and mounting casing) placed in the middle of the rope. The placement of a sensor into the oscillating system represents a defined physical interference (mass loading), which inevitably decreases the natural frequency of the steel wire rope.

**Figure 6:**
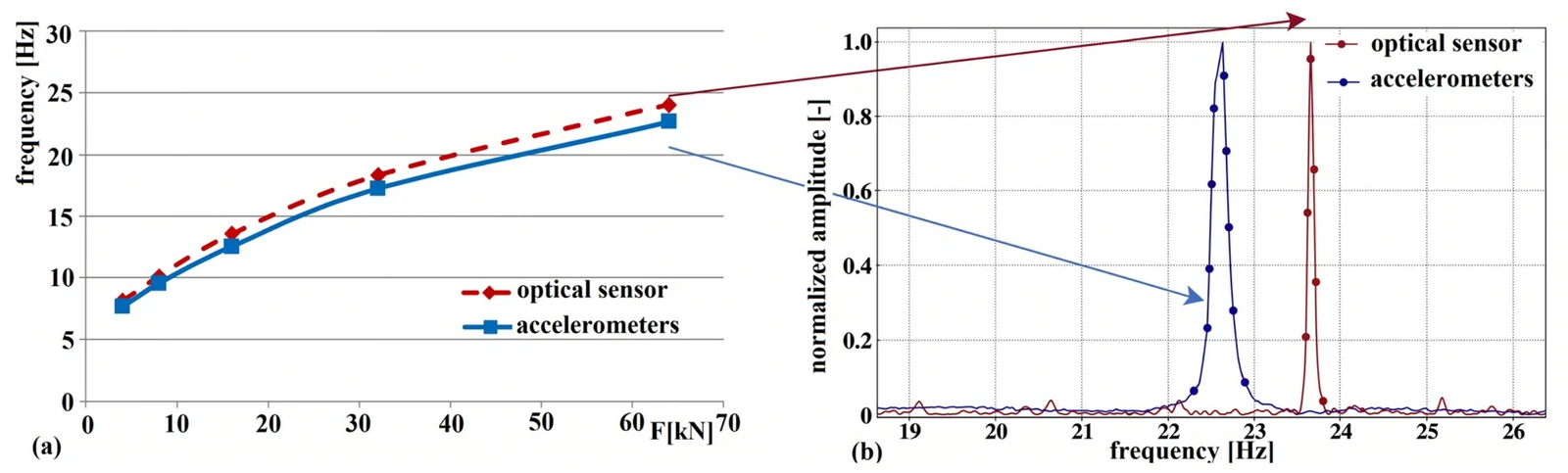
Comparison of measured values using the optical sensor and accelerometers. Measurements performed using the bare rope surface (0 g added mass). (**a**) the trend of change in the fundamental natural frequency of the steel wire rope (Table 1) for the optical sensor (red dashed line) and accelerometers (blue line), (**b**) comparison of the fundamental natural frequency peaks of the steel wire rope (Table 1) at a tension force of 64 kN (optical sensor measurement—red line, accelerometer measurement—blue line).

From the measured data (e.g., 22.631 Hz at 64 kN), it is evident that the accelerometer exhibits lower frequency values (Figure 6, blue line) compared to the values measured by the optical sensor (Figure 6, red dashed line).

**Figure 7:**
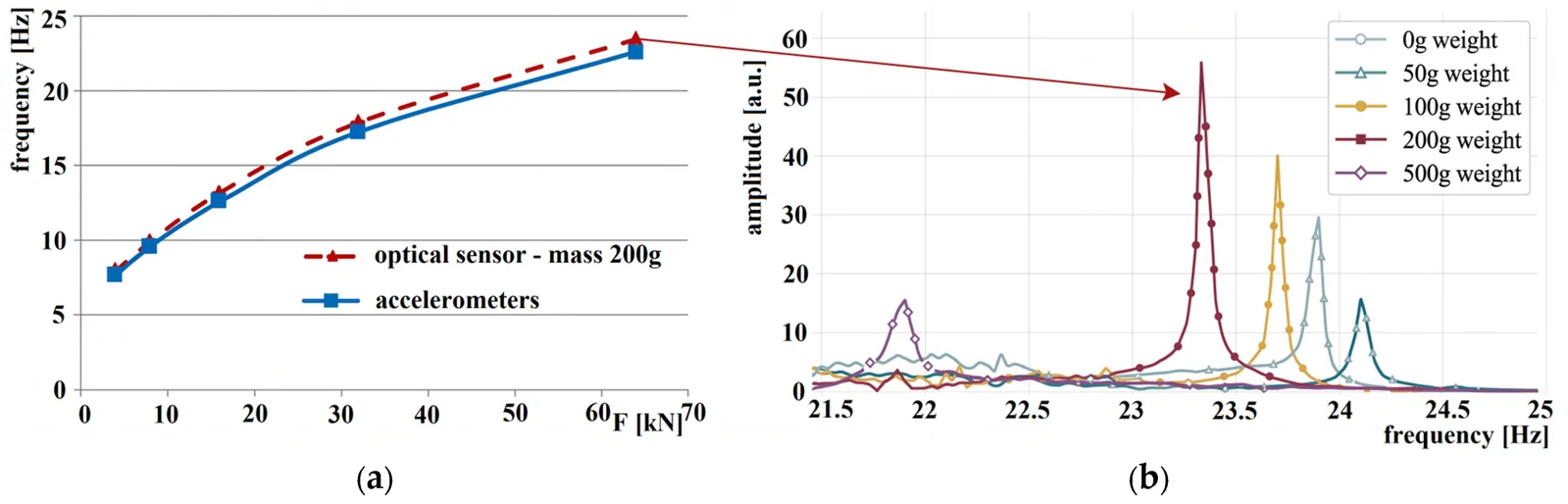
Comparison of the natural frequency values measured by the accelerometer with the natural frequency values measured by the optical sensor with a 200 g suspended mass at tension forces F of 4 kN, 8 kN, 16 kN, 32 kN, and 64 kN. (**a**) comparison of the natural frequency trends (**b**) Spectrum waveforms in the x-axis direction obtained via FFT from the measured Δx values during incremental changes in mass (50 g, 100 g, 200 g, and 500 g) tension force 64 kN.

Figure 7a illustrates the natural frequency trends measured using the 191 g accelerometer assembly (blue line) compared to the natural frequencies measured by the optical sensor with a 200 g point mass suspended from the rope (red dashed line). The trend associated with the 200 g suspended mass exhibits a smaller frequency drop than the 191 g accelerometer assembly. This behavior highlights the dual nature of the mechanical interference caused by contact sensors on lightweight tensioned structures. The rigid clamping mechanism of the accelerometers over a specific section of the rope not only adds an inertial load (mass) but also locally increases the effective bending stiffness. This creates a localized mechanical discontinuity that alters the dynamic response more significantly than a pure point mass of equivalent weight. Consequently, the application of contact accelerometers inherently shifts the dynamic baseline of the system. While a detailed analytical quantification of this localized stiffness variation is beyond the scope of this study, the experimental data demonstrates that the mechanical attachment introduces deterministic boundary-condition effects. The optical approach isolates the measurement from these specific mechanical interferences, enabling an exact evaluation of the rope’s natural dynamics.

## 5. Results and Discussion

### Metrological Sensitivity Bound at Constant Tension

**Figure 8:**
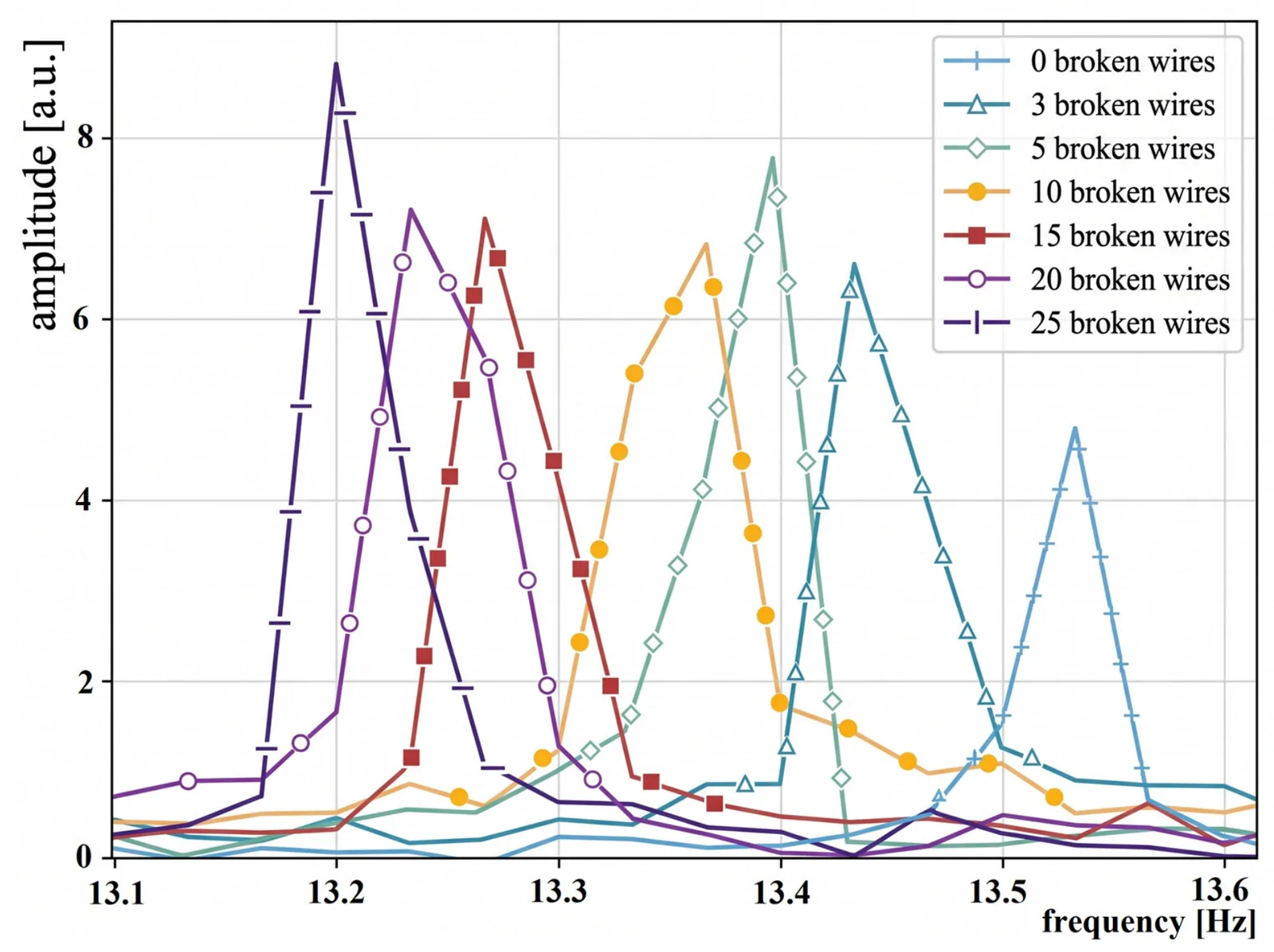
Composite of spectra in the x-axis direction obtained via FFT from measured Δx values at a tension force of 16 kN with wire breaks. Fundamental natural frequency peaks of the rope oscillation, listed progressively from the right: intact rope (no breaks), 3 breaks, 5 breaks, 10 breaks, 15 breaks, 20 breaks, and 25 breaks. Measurements performed using the 28 g reflective plate.

**Figure 9:**
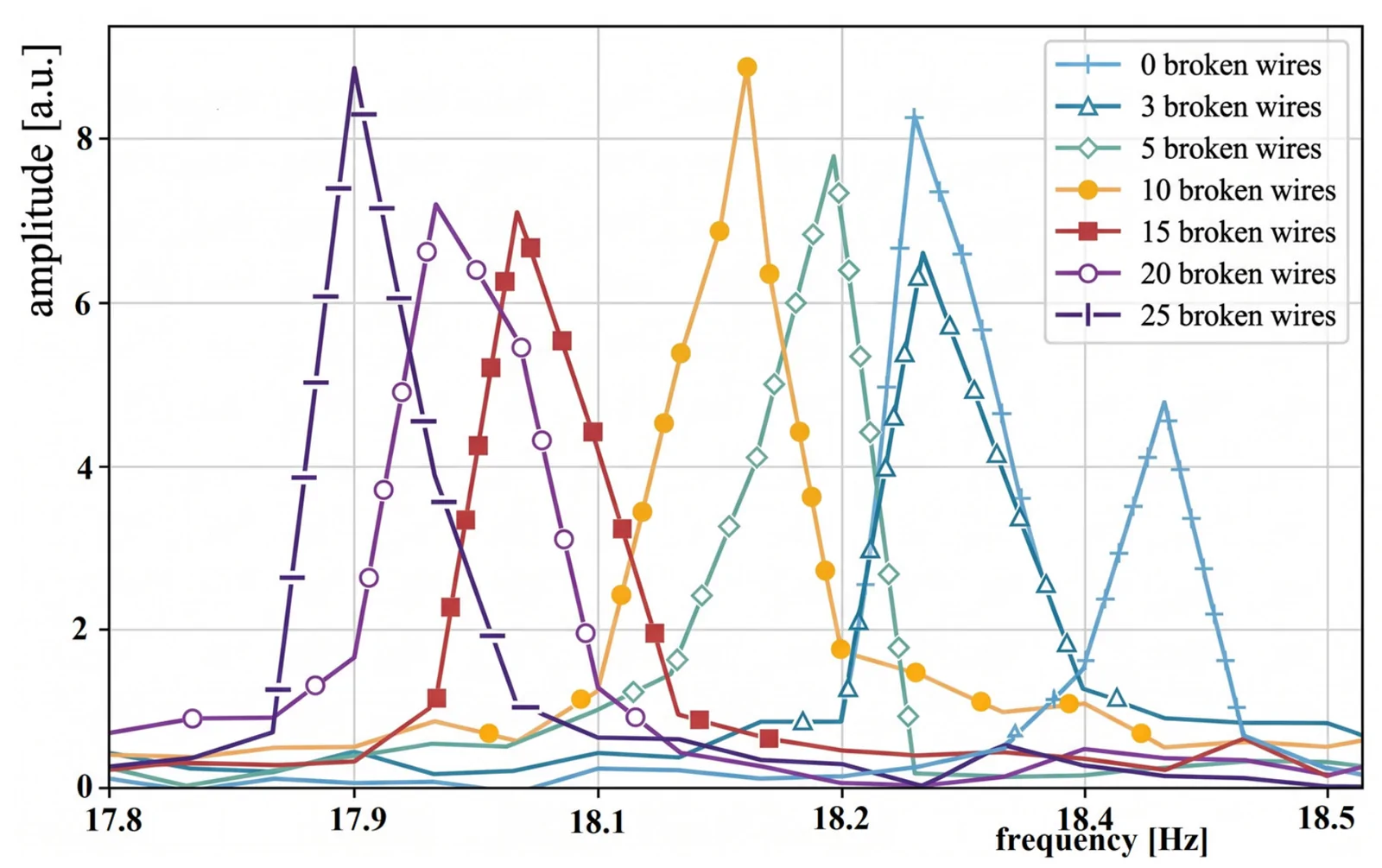
Composite of spectra in the x-axis direction obtained via FFT from measured Δx values at a tension force of 32 kN with induced wire breaks. Fundamental natural frequency peaks of the rope oscillation, listed progressively from the right: intact rope (no breaks), 3 breaks, 5 breaks, 10 breaks, 15 breaks, 20 breaks, and 25 breaks. Measurements were performed using the 28 g reflective plate.

**Figure 10:**
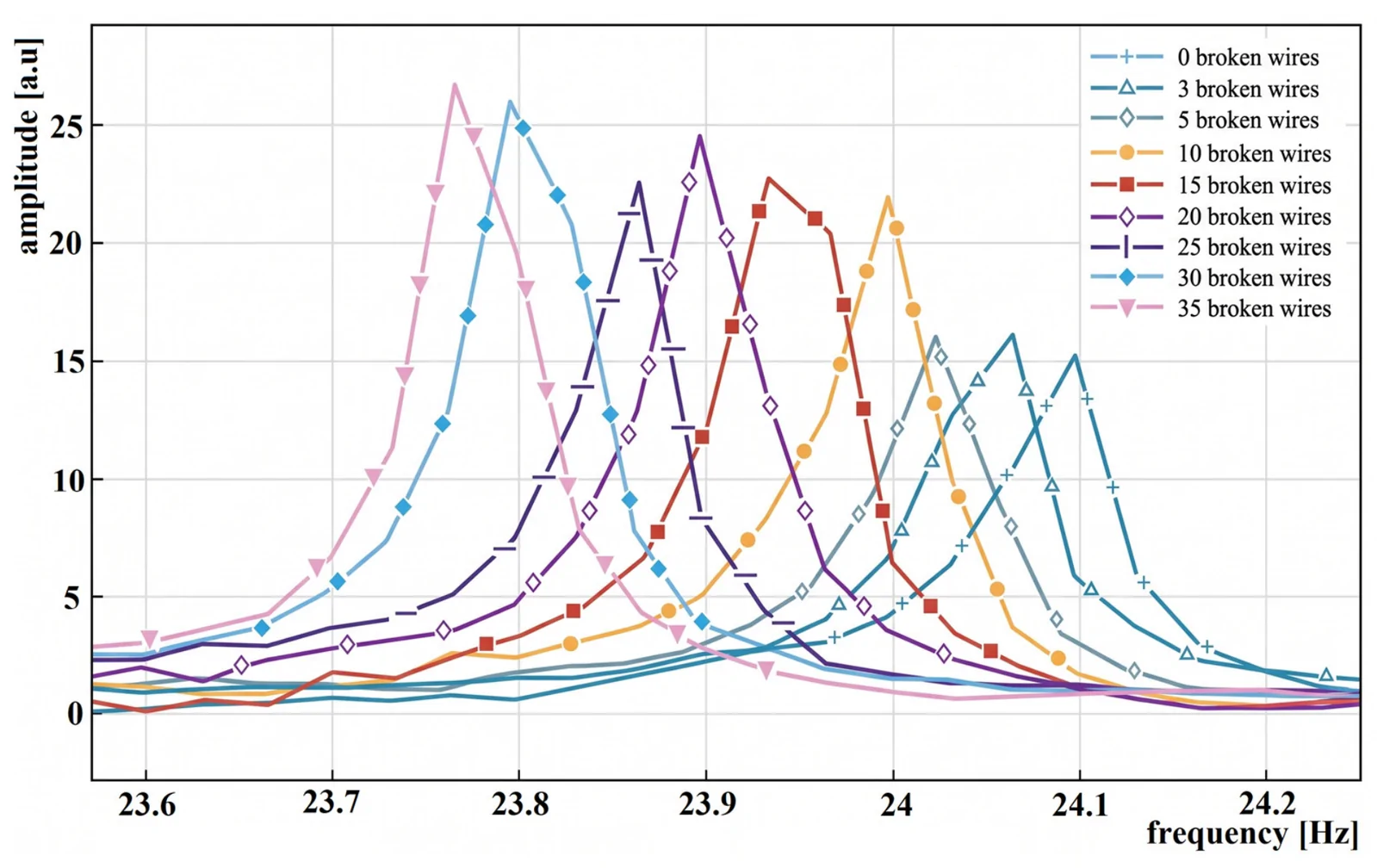
Composite of spectra in the x-axis direction obtained via FFT from measured Δx values at a tension force of 64 kN with induced wire breaks. Fundamental natural frequency peaks of the rope oscillation, listed progressively from the right: intact rope (no breaks), 3, 5, 10, 15, 20, 25, 30, and 35 wire breaks. Measurements were performed using the 28 g reflective plate.

At tension forces of 4 kN and 8 kN, the fundamental natural frequency shifts caused by the local reduction in the metallic cross-section (wire breaks) were in the order of hundredths of Hz. These specific measurements were non-repeatable, likely due to disturbances caused by the manipulation required to create the wire breaks, which altered the measurement conditions—specifically within the rope specimen itself. However, at tension forces of 16 kN (Figure 8), 32 kN (Figure 9), and 64 kN (Figure 10), the spectra in the x-axis direction (obtained via FFT from measured Δx values) show clear and progressive shifts in the fundamental natural frequency corresponding to the induced local defects. At these higher tension levels, the frequency change was detectable even at a local integrity disruption of only three wire breaks, representing a 1.35% local reduction in cross-section. The variations between the individual rope states, caused by the local wire failures, ranged from hundredths to tenths of Hz.

From the overlay of the fundamental natural frequency spectra for a tension force of 16 kN (Figure 8), it is evident that a shift in the natural vibration frequencies occurs during progressive integrity failure compared to the intact rope (Figure 8, blue line). These frequency shifts are in the order of hundredths of Hz. A shift is detectable even with a local disruption of only three wire breaks, which represents a change of 0.1 Hz (Figure 8, blue vs. dark blue line) compared to the intact rope.

Disrupting the rope with five wire breaks causes a frequency shift of 0.136 Hz relative to the intact state (Figure 8, blue vs. green line), while the difference between three and five breaks (Figure 8, dark blue vs. green line) reaches 0.036 Hz. A disruption of ten wire breaks results in a 0.166 Hz shift compared to the intact rope (Figure 8, blue vs. yellow line), with the incremental shift between five and ten breaks being 0.03 Hz. Fifteen wire breaks cause a 0.27 Hz change relative to the intact rope (Figure 8, blue vs. red line). Each subsequent degradation by five additional wire breaks—up to 20 and 25 breaks—shifts the frequency by 0.3 Hz (Figure 8, blue vs. violet line) and 0.33 Hz (Figure 8, blue vs. dark violet line), respectively. Thus, the total frequency shift between the intact specimen and a rope with an 11.26% local loss of metallic cross-section reaches only 0.33 Hz.

The primary cause of the frequency drop upon wire breakage is the loss of axial stiffness (EA) and localized stress redistribution. When wires break, the effective cross-sectional area of the rope decreases. To maintain the same overall tension force (e.g., 64 kN) provided by the testing machine, the remaining intact wires must elongate further, thereby increasing their internal stress. Under the substantial tension of 64 kN, high inter-wire and inter-strand friction forces effectively ‘locked’ the rope structure, preventing any global unraveling or massive strand rearrangement after the cuts. Consequently, stress redistribution resulting from the severed wires remained strictly localized to the immediate vicinity of the defect. This local load redistribution of the material represents a loss of axial stiffness, which reduces the overall dynamic stiffness of the oscillating system. In terms of mass, three broken wires represent a local imbalance of approximately 0.15 g over 10 mm length. Although the reduction in dead mass in an oscillating system generally acts to increase natural frequencies, the recorded downward shift proves the dominance of the loss of axial stiffness. Since the tension force was maintained at a constant level by the testing machine throughout the measurement, the recorded downward shift in the natural frequency is a direct manifestation of the structural degradation. The loss of axial stiffness in this case substantially outweighs the theoretical effect of the mass reduction.

The aforementioned measurements demonstrate the ability of the optical device to sense changes in the rope’s fundamental natural frequency induced by local variations. From a practical perspective, this degree of resolution confirms that the optical method can track the dynamic baseline of the rope without deterministic interference, which is essential for accurate tension monitoring.

**Figure 11:**
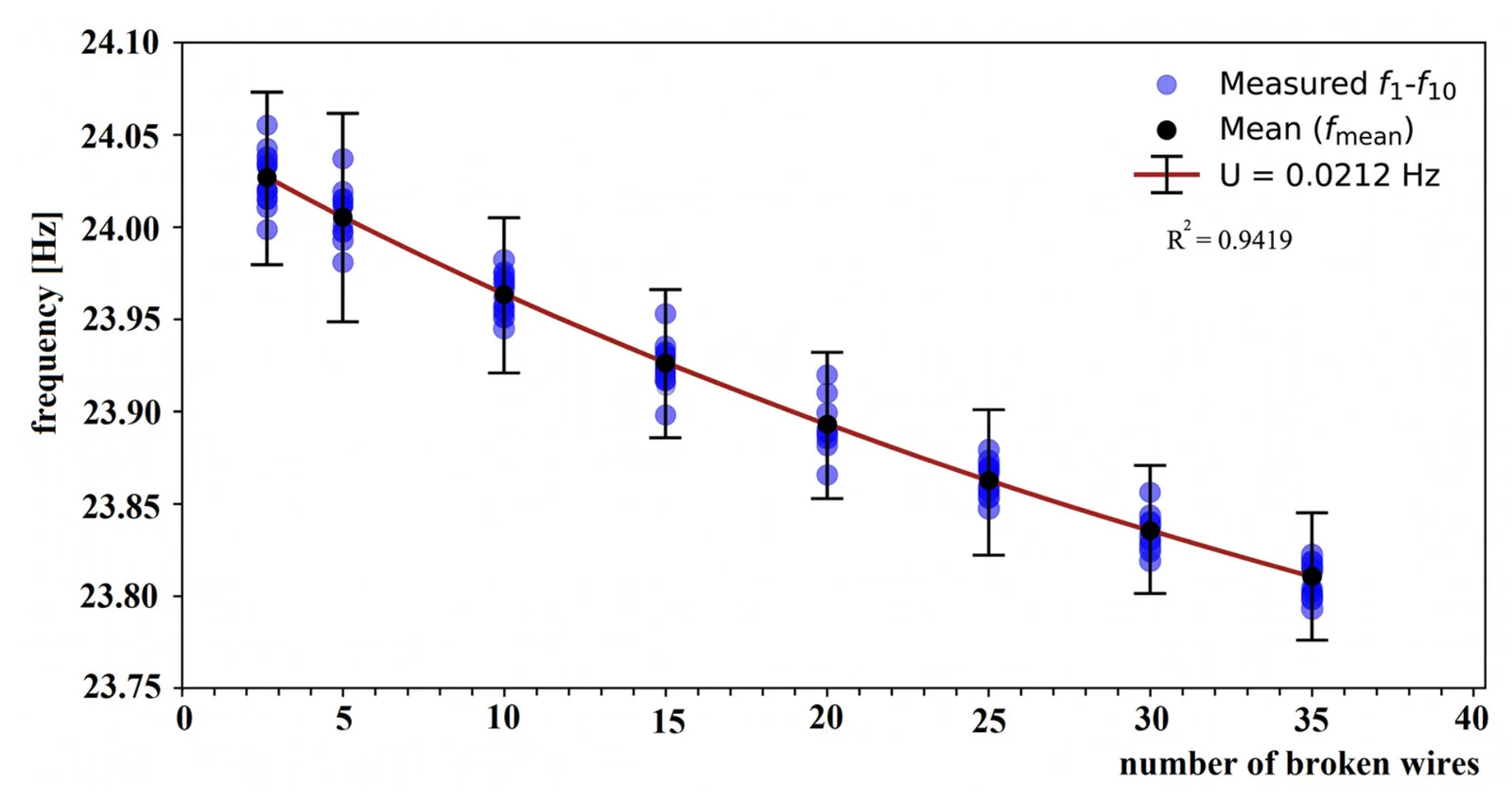
Evaluation of measurement variance in natural frequencies of ten ropes at a 64 kN tensile load during progressive integrity degradation.

To verify the stability and repeatability of the measurement chain, the progressive degradation analysis was performed across the complete set of ten independent rope specimens of identical construction (Section 2). Based on the ten independent rope measurements at each degradation stage under a 64 kN load (Figure 11), the Type A standard uncertainty was determined ($u_{A}=0.0044\;\mathrm{Hz}$). In combination with the Type B uncertainty resulting from spectral resolution ($u_{B}=\Delta f/\sqrt{12}\approx 0.0096\;\mathrm{Hz}$), the total combined standard uncertainty is $u_{c}=0.0106\;\mathrm{Hz}$ (yielding an expanded uncertainty of $U=2\cdot u_{c}=0.0212\;\mathrm{Hz}$ at a 95% confidence level, represented by the error bars in Figure 11). While the Type B uncertainty ($u_{B}=\Delta f/\sqrt{12}\approx 0.0096\;\mathrm{Hz}$) represents a simplified rectangular model of spectral discretization, any additional unmodeled variances induced by the Hann window function, sensor noise, or thermal drift are inherently absorbed and quantified by the experimental Type A uncertainty ($u_{A}=0.0044\;\mathrm{Hz}$) derived from the physical measurements across the ten specimens.

Given that the observed frequency shifts during progressive rope damage range from 0.03 Hz to 0.33 Hz, the measurement method can be considered sufficiently sensitive to reliably capture minute shifts in the system’s dynamic baseline. In metrology, a shift exceeding three times the standard uncertainty—namely 3⋅u_c_ = 0.0318 Hz—is regarded as significant. The smallest observed increment of 0.036 Hz is therefore statistically distinguishable and significant, albeit narrowly.

Linear regression analysis of the measured data at a force of 64 kN demonstrated a direct correlation between the number of broken wires and the decrease in the fundamental natural frequency (R^2^ = 0.9419). From the slope of the regression line, each broken wire in the monitored cross-section under these specific laboratory conditions reduced the natural frequency by an average of 0.0069 Hz. Rather than serving as a universal calibration curve for field NDT, these values establish the metrological sensitivity bound of the proposed optical method under constant tension.

It must be noted that the application of the 28 g reflective target plate inherently introduces a minor mass-loading effect compared to the bare 0 g rope. However, because the plate acts purely as a suspended point mass without mechanically altering the local bending stiffness, its physical contribution manifests strictly as a constant, predictable systematic offset. Consequently, when calculating the actual rope tension based on the fundamental frequency, this minor offset does not distort the underlying physical model. Unlike a rigidly clamped 191 g accelerometer—which would artificially stiffen the rope and introduce non-linear errors into the tension calculation—the non-contact optical approach with a 28 g target preserves the metrological validity of the evaluated tension values.

## 6. Conclusions

Based on the theoretical modeling, evaluation of boundary conditions, and metrological sensitivity validation of the experimental data, the following conclusions are drawn:

Necessity of an advanced theoretical baseline: While the simplified ideal string equation is suitable for basic global dynamic estimations, capturing localized structural degradation at constant tension requires the full beam-string model. Incorporating the structural geometric factor ($G_{m}$) is essential to properly account for the dynamic bending stiffness of the 6×37 FC wire rope.

Quantification of contact sensor interference: The application of a conventional contact accelerometer (191 g assembly) introduces a deterministic inertial load (mass-loading effect) and locally alters the bending stiffness. This physical interference distorts the natural frequency baseline, limiting the accuracy required to isolate minute frequency shifts caused by early-stage structural changes.

Metrological sensitivity of the optical methodology: The proposed non-contact optical flow sensor methodology avoids these deterministic contact-induced errors. Supported by a repeatability analysis across multiple specimens, the measurement chain achieved a combined standard uncertainty of $u_{c}=0.0106\;\mathrm{Hz}$, confirming that frequency shifts as small as 0.036 Hz are statistically distinguishable.

Mitigation of optical surface constraints: To transition optical flow tracking from ideal conditions to controlled laboratory applications, the use of a rigid lightweight reflective plate (28 g) proved effective. It eliminates optical artifacts generated by the rope’s 3D helical surface geometry and maintains the required focal depth (lift-off distance) during transverse oscillations.

Dominance of axial stiffness reduction: The localized reduction in the steel rope’s load-bearing cross-section induces local stress redistribution. Despite the minor theoretical mass reduction, the macroscopic dynamic manifestation is a downward shift in the natural frequency (averaging 0.0069 Hz per broken wire at 64 kN), demonstrating that the loss of axial stiffness is the primary driver of the system’s dynamic response.

Scope of laboratory verification: While the controlled wire-cut tests under laboratory conditions do not constitute a proven field NDT method for quantitative damage counting, the demonstrated linear correlation ($R^{2}=0.9419$) confirms the metrological sensitivity bound of the optical setup. When utilized for absolute rope tension assessment, the methodology requires prior knowledge or calibration of baseline physical parameters—specifically the mass per unit length (μ), effective vibrating length (L), boundary conditions, and load-dependent Effective rope modulus ($E_{\mathrm{eff}}$).

The primary practical value of this approach lies in providing a minimally invasive baseline monitoring framework for controlled environments without the mechanical interference inherent to contact sensors.

## Figures and Tables

**Table 3:** Measurement system technical specification.

Category	Parameter	Nominal Value
Sensor	Technology Type	HERO 25K
Resolution	Configured DPI	25,600 DPI
Sampling	Frequency (Polling)	1000 Hz
Response	Latency (1/f)	1.0 ms
Sensitivity	Spatial Step (L)	0.992 µm
Dynamics	Max. Velocity (v_max)_	>10.16 m·s^−1^
Dynamics	Max. Acceleration (a_max_)	>392 m·s^−2^
Accuracy	Theoretical Linearity	<1.0%

**Table 4:** Hardware and Software Specifications.

Category	Parameter/Function	Specification and Value
Hardware/Connectivity	Connectivity Supported device	HID (USB)—Vendor ID: 0x046d, Product ID: 0xc099
Sampling	Sampling frequency (Fs)	1000 Hz (1ms sample interval)
	Fs threshold monitoring	Warning triggered below 450 Hz
	Acquisition time	30 s
	Calibration	10 s duration (for noise and offset elimination)
Signal Processing	Filter type (Displacement)	5th-order Butterworth (SOS implementation)
	Filter frequency range	1 Hz to 200 Hz (High-pass/Low-pass)
	Smoothing	Moving average, 20-sample window
Acceleration Analysis	Calculation method	Numerical gradient of velocity (Delta data)
	Acceleration filter	Butterworth Low-pass, cutoff 100 Hz
Spectral Analysis	Displayed range (FFT)	0 Hz to 200 Hz (max. 250 Hz per Nyquist)
	Transform type	Single full-take DFT (Hann window)
	Native bin resolution	Δf ≈ 0.033 Hz
	Envelope analysis	Hilbert transform (shock and modulation detection)
Reporting	Output format	Interactive HTML (Plotly engine ver. 6.3.0)
	Analytical modes	SINGLE (detailed diagnostics)/BATCH (comparison)

## Data Availability

The raw data supporting the conclusions of this article will be made available by the authors on request.
